# Ground beetles (Coleoptera: Carabidae) of rice field banks and restored habitats in an agricultural area of the Po Plain (Lombardy, Italy)

**DOI:** 10.3897/BDJ.1.e972

**Published:** 2013-11-11

**Authors:** Nicola Pilon, Elisa Cardarelli, Giuseppe Bogliani

**Affiliations:** † Elitron, via Capri 11/3, 20153 Milano, Italy; ‡ Dept. of Earth and Environmental Sciences, University of Pavia, Via Ferrata 9, 27100 Pavia, Italy

**Keywords:** Carabidae, agroecosystem, rice fields, habitat restoration, Italy, Po plain

## Abstract

An entomological investigation was carried out in an agricultural area, mainly rice fields, of the Po river plain, located in the municipalities of Lacchiarella (MI) and Giussago (PV) (Lombardy, Italy). In 2009 and 2010, ground beetles (Coleoptera: Carabidae) were sampled along rice field banks and in restored habitats, by means of pitfall traps. The area appeared as species-rich, compared to other anthropogenic habitats in the Po river pain. Most of the collected Carabids were species with a wide distribution in the Paleartic region, eurytopic and common in European agroecosystems. The assemblages were dominated by small-medium, macropterous species, with summer larvae. No endemic species were found. Species with southern distribution, rarely found north of the Po river, were also sampled. *Amara littorea* is recorded for the first time in Italy.

## Introduction

In the last decades, intensification and mechanization of agricultural practices, introduced in order to maximise productivity, led to a decrease in habitat quality and landscape heterogeneity throughout European agroecosystems. Diffusion of monoculture, increased use of chemicals (i.e. pesticides and fertilizers) and removal of non-cropped areas, like small woodlots and hedges, caused a wide-scale loss of biodiversity (Stoate et al. 2001).

Recently, environmentally-friendly agronomic practices and creation of non-cropped habitats have been recognized as a potential solution to this dramatic decline of biodiversity and have become key aims of European Union’s Common Agricultural Policy (CAP) and, as a consequence, of national and regional ones (Stoate et al. 2009). In Lombardy lowland, environmentally-friendly measures includes reforestations, creation of hedges and buffer strips, maintenance of meadows and renaturalization of wetlands (Lombardy Region 2012, http://www.agricoltura.regione.lombardia.it).

Even if agri-environment schemes (AESs) benefit some farmland species (e.g., Peach et al. 2001), gaps in the provision of habitat quality and landscape connectivity for many others still exist (Kleijn et al. 2001, Vickery et al. 2004, Reid et al. 2007). Better understanding on effects of AESs on farmland biodiversity and exhaustive surveys on animal and plant communities in enhanced habitats are required (Kleijn and Sutherland 2003, Stoate et al. 2009).

The aim of this research was to investigate the Carabid assemblages of an intensive agricultural area (mainly rice fields) subjected to environmental improvements since 1996, in particular the creation of buffer strips along paddy fields and the restoration of an area of ​​150 ha.

## Materials and methods

### Study area

**Figure 1.**
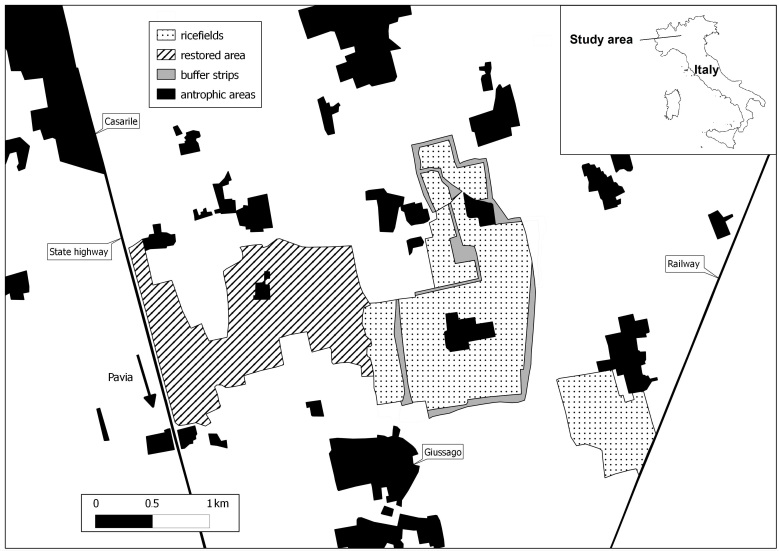
Schematic representation of the study area (anthropic areas include villages, farmsteads, main roads and railways).

The study was carried out in an 4.5 km^2^ agricultural area, mainly cultivated with rice, located in north-western Italy, in the middle of the Po plain, approximately 13 km north from the city of Pavia, in the municipalities of Lacchiarella (MI) and Giussago (PV); barycentre 45°17'38.63"N, 09°08'52.08"E (Fig. 1).

**Figure 2.**
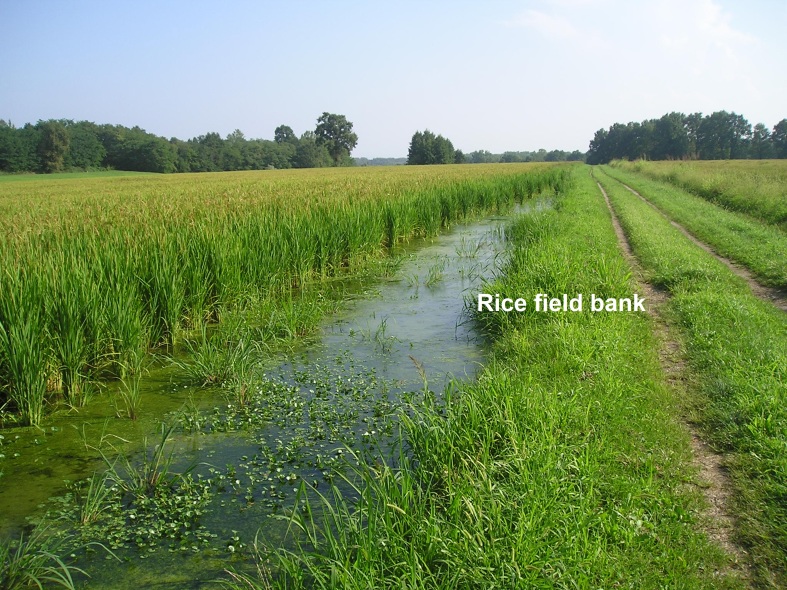
Rice field with herbaceous banks.

**Figure 3.**
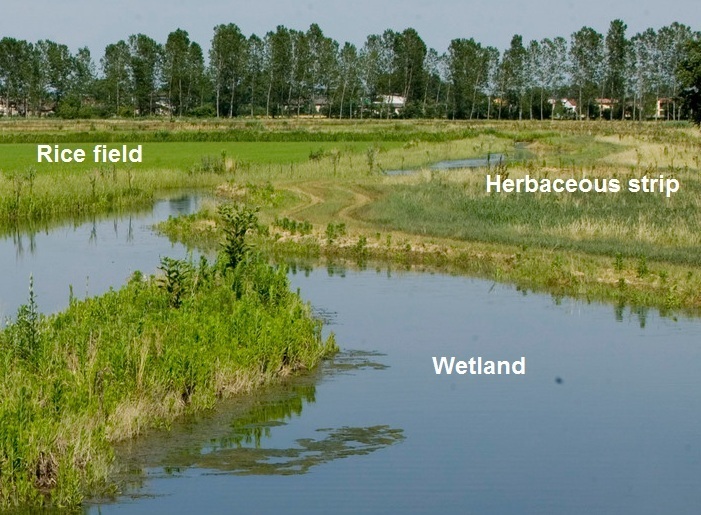
Herbaceous buffer strip along a small wetland connected to paddy field.

**Figure 4.**
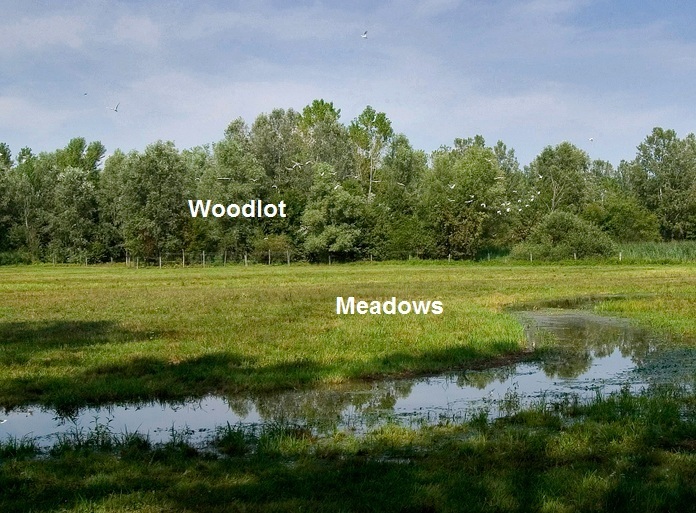
Wet meadow with reforested area on the background.

The study area included three adjacent rice farms, “La Darsena”, “La Cadenazza” and “Necchi”, and a restored area, “La Cassinazza”. The Carabid fauna was sampled in:

- *rice field banks* (Fig. 2): characterized by herbaceous cover (mainly *Setaria glauca*, *Carex elata*, *Avena sativa*, *Convolvolus arvensis*, *Trifolium pratense* and *Lolium perenne*), sporadically with a row of poplar trees (*Populus canadensis*);
- *buffer strips* (Fig. 3): perimeter land of paddy fields taken out of production and converted into small wetlands and strips of permanent vegetation, planted with autochthonous shrubs and trees (mainly *Quercus robur*, *Carpinus betulus*, *Fraxinus angustifolia*, *Crataegus monogyna*, *Prunus spinosa* and *Salix cinerea*). The first stands were planted in 2003 and, during the study period, strips were fully-developed into arboreal habitats (*arboreal buffer strips*). The last stands were planted in 2009 and, during the study period, strips were mostly covered by herbaceous vegetation (*herbaceous buffer strips*, mainly *Echinochloa crus-galli*, *Polygonum minus*, *Setaria glauca*, *Lolium perenne*, *Chenopodium album* and *Humulus lupulus*);
- *restored area* (~150 ha; Fig. 4): formerly a farmland area undergoing restoration since 1996. The area is composed by a mosaic of different habitats, including wetlands, reforested areas and meadows, connected by a system of hedges. For the descriptive purposes of this paper, Carabid coenosis of meadows, both wet and dry (*herbaceous restored habitats*, mainly *Convolvolus arvensis*, *Lolium perenne*, *Lotus corniculatus*, *Trifolium pratense*, *Solidago gigantea*, *Bidens tripartita* and *Taraxacum officinale*), was divided from that of the forested areas and hedges (*arboreal restored habitats*, mainly *Quercus robur*, *Salix alba*, *Carpinus betulus*, *Alnus glutinosa*, *Ulmus campestris*, *Populus tremula*, *Fraxinus angustifolia*, *Crataegus monogyna*, *Prunus spinosa*, *Viburnus opulus* and *Salix cinerea*).

### Sampling method and data analysis

Ground beetles were sampled using plastic pitfall traps (62 mm in diameter and 70 mm deep) buried in the soil and filled with 50 ml of wine vinegar and a drop of detergent (Brandmayr et al. 2005). Pitfalls were covered with a 10 × 10 cm wooden roof to prevent flooding and emptied fortnightly.

Along rice field banks, we placed a total of 60 traps from April to November 2009 and 68 traps from May to November 2010; along buffer strips, we positioned 56 traps from May to November 2009 and 2010; in the restored area, we placed 66 traps from July to November 2009 and from April to November 2010.

Carabids were identified to the species level following the nomenclature of *Fauna Europaea* (http://www.faunaeur.org, Vigna-Taglianti 2010). Information on chorotype, body size, larval and wing development were reported for each species. Chorotype were obtained from Vigna-Taglianti 2005; larval development were derived from Casale et al. 1982, Drioli 1987 and Brandmayr et al. 2005; data on body size and wing development were mainly obtained from Hůrka 1996, and secondly from Jeannel 1941, Jeannel 1942. As for body size, according to Cole et al. 2002, species were divided as (a) very small (< 5 mm), (b) small (5 - 9 mm), (c) medium (9 - 15 mm) and (d) large (> 15 mm). Data on adult diet were not available for all species and we reported only the existing information, according to Cole et al. 2002, Brandmayr et al. 2005, Purtauf et al. 2005, Melis et al. 2010 and Bettacchioli et al. 2012.

A synthetic description of habitat preference, derived from Hůrka 1996 and personal observations with special reference to the Po plain, were also reported for each species. According to Fournier and Loreau (1999), we classified the species as "rare" when the total capture over the whole area was lower than 0.1% (i.e. < 35 individuals); the other species were classified as "common" and the two most abundant species as "dominant". The total number of captured individuals (*n*) was reported in brackets.

As for rice field banks and enhanced habitats, ground beetle abundances were expressed both as absolute frequency (i.e. number of collected individuals) and as annual Activity Density (*aAD*; Brandmayr et al. 2005), that is the number of collected individuals during the entire sampling period (*n_tot_*) divided by sampling effort (*US*) for each sampling station:

DAa = n_tot_ / US

with *US = Σ us* and us = trap * (gg/10), where *trap* is number of traps and *gg* is the number of days during which the traps were active in each sampling session (Suppl. material 1).

Specimens, dried or preserved in alcohol, are stored in the author’s collections (Nicola Pilon, Milano) and in the collection of the University of Pavia.

## Checklists

### Checklist

#### Acinopus picipes

(Olivier, 1795)

##### Notes

Turanic-European. Open habitats, thermophilous. Macropterous, with winter larvae. Medium size. Spermatophagous.

Uncommon north of the Po river. Rare in the study area (*n* = 2); recorded in arboreal restored habitats only.

#### Acupalpus elegans

(Dejean, 1829)

##### Notes

Turanic-European-Mediterranean. Open habitats, halophilous. Macropterous, with summer larvae. Very small size. Spermatophagous.

Rare in the study area (*n* = 1); recorded in herbaceous restored habitats only.

#### Acupalpus flavicollis

(Sturm, 1825)

##### Notes

European. Paludicolous, ripicolous. Macropterous, with summer larvae. Very small size. Spermatophagous.

Rare in the study area (*n* = 1); recorded in arboreal restored habitats only.

#### Acupalpus maculatus

(Schaum, 1860)

##### Notes

European-Mediterranean. Paludicolous, ripicolous. Macropterous, with summer larvae. Very small size. Spermatophagous.

Rare in the study area (*n* = 18).

#### Acupalpus notatus

Mulsant

##### Notes

Mediterranean. Paludicolous, halophilous. Macropterous, with summer larvae. Very small size. Spermatophagous.

Rare in the study area (*n* = 1); recorded in arboreal restored habitats only.

#### Agonum emarginatum

(Gyllenhal, 1827)

##### Notes

European. Paludicolous, ripicolous. Macropterous, with summer larvae. Small size.

Common in the study area (*n* = 107). Recorded in all habitat categories.

#### Agonum muelleri

(Herbst, 1784)

##### Notes

Siberic-European (Holoartic). Open habitats, hygrophilous. Macropterous, with summer larvae. Small size. Predator.

Rare in the study area (*n* = 8).

#### Agonum sexpunctatum

(Linné, 1758)

##### Notes

Siberic-European. Open habitats, hygrophilous. Macropterous, with summer larvae. Small size. Predator.

Rare in the study area (*n* = 1); recorded in rice field banks only.

#### Agonum versutum

Sturm, 1824

##### Notes

Siberic-European. Paludicolous, silvi-ripicolous. Macropterous, with summer larvae. Small size.

Rare in the study area (*n* = 2); recorded in arboreal restored habitats only.

#### Agonum viduum

(Panzer, 1796)

##### Notes

Siberic-European. Paludicolous, silvi-ripicolous. Macropterous, with summer larvae. Small size.

Rare in the study area (*n* = 8); recorded in arboreal restored habitats only.

#### Amara aenea

(De Geer, 1774)

##### Notes

Paleartic (Holoartic). Open habitats, eurytopic. Macropterous, with summer larvae. Small size. Zoospermatophagous.

Common in the study area (*n* = 1180). Recorded in all habitat categories.

#### Amara bifrons

(Gyllenhal, 1810)

##### Notes

Central Asiatic-European. Open habitats. Macropterous, with winter larvae. Small size. Zoospermatophagous.

Rare in the study area (*n* = 2).

#### Amara communis

(Panzer, 1797)

##### Notes

Asiatic-European. Open habitats, hygrophilous. Macropterous, with summer larvae. Small size. Zoospermatophagous.

Rare in the study area (*n* = 7).

#### Amara familiaris

(Duftschmid, 1812)

##### Notes

Siberic-European. Open habitats, eurytopic. Macropterous, with summer larvae. Small size. Zoospermatophagous.

Rare in the study area (*n* = 10).

#### Amara fulvipes

(Audinet-Serville, 1821)

##### Notes

European. Open habitats, xerophilous. Macropterous, with summer larvae. Medium size. Zoospermatophagous.

Rare in the study area (*n* = 4).

#### Amara littorea

C.G. Thomson, 1857

##### Notes

Asiatic-European. Open habitats, xerophilous. Macropterous, with summer larvae. Small size. Zoospermatophagous.

Recorded with certainty for the first time in Italy (Cardarelli and Pilon 2012). Rare in the study area (*n* = 1); recorded in herbaceous buffer strips only.

#### Amara lucida

(Duftschmid, 1812)

##### Notes

Turanic-European. Open habitats, xerophilous. Macropterous, with summer larvae. Small size. Zoospermatophagous.

Rare in the study area (*n* = 31).

#### Amara nitida

Sturm, 1825

##### Notes

Asiatic-European. Open habitats. Macropterous, with summer larvae. Small size. Zoospermatophagous.

Rare in the study area (*n* = 12); recorded in rice field banks only.

#### Amara similata

(Gyllenhal, 1810)

##### Notes

Asiatic-European. Open habitats, eurytopic. Macropterous, with summer larvae. Small size. Zoospermatophagous.

Common in the study area (*n* = 203).

#### Amblystomus niger

(Heer, 1841)

##### Notes

European-Mediterranean. Open habitats, thermophilous. Macropterous, with summer larvae. Very small size.

Uncommon north of the Po river. Rare in the study area (*n* = 7).

#### Anchomenus dorsalis

(Pontoppidan, 1763)

##### Notes

Paleartic. Open habitats, hygrophilous. Macropterous, with summer larvae. Small size. Predator.

Common in the study area (*n* = 234). Recorded in all habitat categories.

#### Anisodactylus binotatus

(Fabricius, 1787)

##### Notes

Asiatic-European. Open habitats, eurytopic. Macropterous, with summer larvae. Medium size. Zoospermatophagous.

Common in the study area (*n* = 761). Recorded in all habitat categories.

#### Anisodactylus signatus

(Panzer, 1796)

##### Notes

Asiatic-European. Open habitats, hygrophilous. Macropterous, with summer larvae. Medium size. Zoospermatophagous.

Common in the study area (*n* = 798). Recorded in all habitat categories.

#### Badister bullatus

(Schrank, 1798)

##### Notes

Holoartic. Open habitats. Macropterous, with summer larvae. Small size. Predator.

Rare in the study area (*n* = 11).

#### Badister sodalis

(Duftschmid, 1812)

##### Notes

Turanic-European. Paludicolous, silvi-ripicolous. Macropterous, with summer larvae. Very small size. Predator.

Rare in the study area (*n* = 1); recorded in arboreal buffer strips only.

#### Bembidion quadrimaculatum

(Linné, 1761)

##### Notes

Holoartic. Open habitats, hygrophilous. Macropterous, with summer larvae. Very small size. Zoospermatophagous.

Common in the study area (*n* = 866). Recorded in all habitat categories.

#### Bembidion quadripustulatum

Audinet-Serville, 1821

##### Notes

Central Asiatic-European-Mediterranean. Open habitats, hygrophilous. Macropterous, with summer larvae. Very small size.

Rare in the study area (*n* = 5); recorded in herbaceous buffer strips only.

#### Brachinus elegans

Chaudoir, 1842

##### Notes

Mediterranean. Open habitats, hygrophilous. Macropterous, with summer larvae. Small size.

Common in the study area (*n* = 1001). Recorded in all habitat categories.

#### Brachinus explodens

Duftschmid, 1812

##### Notes

Asiatic-European. Open habitats. Macropterous, with summer larvae. Small size. Predator.

Rare in the study area (*n* = 2).

#### Brachinus glabratus

Latreille

##### Notes

S-European. Open habitats, hygrophilous. Macropterous, with summer larvae. Small size.

Rare in the study area (*n* = 14).

#### Brachinus plagiatus

Reiche, 1868

##### Notes

Mediterranean. Open habitats, halophilous. Macropterous, with summer larvae. Small size. Predator.

Rare in the study area (*n* = 26).

#### Brachinus sclopeta

(Fabricius, 1792)

##### Notes

European-Mediterranean. Open habitats, hygrophilous. Macropterous, with summer larvae. Small size. Predator.

Common in the study area (*n* = 372).

#### Bradycellus verbasci

(Duftschmid, 1812)

##### Notes

Turanic-European. Open habitats, xerophilous. Macropterous, with winter larvae. Very small size. Spermatophagous.

Rare in the study area (*n* = 2); recorded in herbaceous buffer strips only.

#### Calathus fuscipes

(Goeze, 1777)

##### Notes

European-Mediterranean. Open habitats, xerophilous. Pteridimorphic, with winter larvae. Medium size. Predator.

Rare in the study area (*n* = 8).

#### Calathus melanocephalus

(Linné, 1758)

##### Notes

Paleartic. Open habitats, xerophilous. Pteridimorphic, with winter larvae. Small size. Predator.

Common in the study area (*n* = 177).

#### Calosoma auropunctatum

(Herbst, 1784)

##### Notes

Central Asiatic-European. Open habitats, xerophilous. Macropterous, with summer larvae. Large size. Predator.

Common in the study area (*n* = 115).

#### Carabus granulatus

Linné, 1758

##### Notes

Asiatic-European (Holoartic). Paludicolous, silvi-ripicolous. Pteridimorphic, with summer larvae. Large size. Predator.

Common in the study area (*n* = 64).

#### Chlaeniellus nitidulus

(Schrank, 1781)

##### Notes

Central Asiatic-European. Paludicolous. Macropterous, with summer larvae. Medium size.

Common in the study area (*n* = 123). Recorded in all habitat categories.

#### Chlaeniellus tristis

(Schaller, 1783)

##### Notes

Paleartic. Paludicolous. Macropterous, with summer larvae. Medium size. Predator.

Rare in the study area (*n* = 4).

#### Chlaenius spoliatus

(P. Rossi, 1792)

##### Notes

Paleartic. Paludicolous. Macropterous, with summer larvae. Large size. Predator.

Common in the study area (*n* = 62). Recorded in all habitat categories.

#### Cicindela campestris

Linné, 1758

##### Notes

Paleartic. Open habitats. Macropterous, with poliennal larvae. Medium size. Predator.

Rare in the study area (*n* = 1); recorded in rice field banks only.

#### Clivina collaris

(Herbst, 1784)

##### Notes

Turanic-European. Open habitats, hygrophilous. Macropterous, with summer larvae. Small size.

Rare in the study area (*n* = 1); recorded in rice field banks only.

#### Clivina fossor

(Linné, 1758)

##### Notes

Asiatic-European (Holoartic). Open habitats, hygrophilous. Pteridimophic, with summer larvae. Small size. Predator.

Rare in the study area (*n* = 28). Recorded in all habitat categories.

#### Diachromus germanus

(Linné, 1758)

##### Notes

Turanic-European-Mediterranean. Open habitats. Macropterous, with summer larvae. Small size.

Common in the study area (*n* = 161).

#### Dinodes decipiens

(L. Dufour, 1820)

##### Notes

European-Mediterranean. Open habitats, xerophilous. Macropterous, with summer larvae. Medium size.

Uncommon north of the Po river. Rare in the study area (*n* = 3).

#### Dolichus halensis

(Schaller, 1783)

##### Notes

Asiatic-European. Open habitats. Macropterous, with winter larvae. Large size.

Rare in the study area (*n* = 5); recorded in herbaceous buffer strips only.

#### Drypta dentata

(P. Rossi, 1790)

##### Notes

Afrotropical and Paleartic. Paludicolous. Macropterous, with summer larvae. Small size. Predator.

Rare in the study area (*n* = 1); recorded in arboreal buffer strips only.

#### Harpalus affinis

(Schrank, 1781)

##### Notes

Asiatic-European (Holoartic). Open habitats. Macropterous, with summer larvae. Medium size. Zoospermatophagous.

Common in the study area (*n* = 372).

#### Harpalus albanicus

Reitter, 1900

##### Notes

S-European. Open habitats. Macropterous, with summer larvae. Small size. Zoospermatophagous.

Rare in the study area (*n* = 3); recorded in rice field banks only.

#### Harpalus anxius

(Duftschmid, 1812)

##### Notes

Paleartic. Open habitats. Macropterous, with summer larvae. Small size. Zoospermatophagous.

Common in the study area (*n* = 331). Recorded in all habitat categories.

#### Harpalus cupreus

Dejean, 1829

##### Notes

S-European. Open habitats, thermophilous. Macropterous, with summer larvae. Medium size. Zoospermatophagous.

Uncommon north of the Po river. Rare in the study area (*n* = 12).

#### Harpalus dimidiatus

(P. Rossi, 1790)

##### Notes

European. Open habitats, xerophilous. Macropterous, with summer larvae. Medium size. Zoospermatophagous.

Rare in the study area (*n* = 15).

#### Harpalus distinguendus

(Duftschmid, 1812)

##### Notes

Paleartic. Open habitats. Macropterous, with summer larvae. Medium size. Zoospermatophagous.

Common in the study area (*n* = 1396). Recorded in all habitat categories.

#### Harpalus luteicornis

(Duftschmid, 1812)

##### Notes

European. Open habitats, hygrophilous. Macropterous, with summer larvae. Small size. Zoospermatophagous.

Common in the study area (*n* = 80). Recorded in all habitat categories.

#### Harpalus oblitus

Dejean, 1829

##### Notes

Turanic-European-Mediterranean. Open habitats, xerophilous. Macropterous, with summer larvae. Medium size. Zoospermatophagous.

Uncommon north of the Po river. Common in the study area (*n* = 61).

#### Harpalus pumilus

Sturm, 1818

##### Notes

Paleartic. Open habitats, xerophilous. Pteridimorphic, with summer larvae. Small size. Zoospermatophagous.

Rare in the study area (*n* = 2); recorded in rice field banks only.

#### Harpalus pygmaeus

Dejean, 1829

##### Notes

S-European. Open habitats. Macropterous, with summer larvae. Small size. Zoospermatophagous.

Common in the study area (*n* = 51).

#### Harpalus rubripes

(Duftschmid, 1812)

##### Notes

Asiatic-European. Open habitats. Macropterous, with summer larvae. Medium size. Zoospermatophagous.

Common in the study area (*n* = 180). Recorded in all habitat categories.

#### Harpalus serripes

(Quensel in Schönherr, 1806)

##### Notes

Paleartic. Open habitats, xerophilous. Macropterous, with summer larvae. Medium size. Zoospermatophagous.

Common in the study area (*n* = 177).

#### Harpalus tardus

(Panzer, 1797)

##### Notes

Asiatic-European. Open habitats, eurytopic. Macropterous, with summer larvae. Medium size. Zoospermatophagous.

Common in the study area (*n* = 97).

#### Limodromus assimilis

(Paykull, 1790)

##### Notes

Siberic-European. Paludicolous, silvi-ripicolous. Macropterous, with summer larvae. Medium size. Predator.

Common in the study area (*n* = 263); recorded in arboreal restored habitats only.

#### Limodromus krynickii

(Sperk, 1835)

##### Notes

Siberic-European. Paludicolous, silvi-ripicolous. Macropterous, with summer larvae. Medium size.

Common in the study area (*n* = 50); recorded in arboreal restored habitats only.

#### Metallina lampros

(Herbst, 1784)

##### Notes

Paleartic (Holoartic). Open habitats, eurytopic. Pteridimorphic, with summer larvae. Very small size. Predator.

Common in the study area (*n* = 49).

#### Metallina properans

(Stephens, 1828)

##### Notes

Siberic-European. Open habitats, eurytopic. Pteridimorphic, with summer larvae. Very small size.

Common in the study area (*n* = 225). Recorded in all habitat categories.

#### Microlestes corticalis

(L. Dufour, 1820)

##### Notes

Turanic-Mediterranean. Open habitats. Macropterous, with summer larvae. Very small size.

Rare in the study area (*n* = 2).

#### Microlestes minutulus

(Goeze, 1777)

##### Notes

Holoartic. Open habitats, eurytopic. Pteridimorphic, with summer larvae. Very small size.

Common in the study area (*n* = 111).

#### Nebria brevicollis

(Fabricius, 1792)

##### Notes

Turanic-European. Open habitats, hygrophilous. Macropterous, with winter larvae. Medium size. Predator.

Rare in the study area (*n* = 1); recorded in arboreal restored habitats only.

#### Oodes helopioides

(Fabricius, 1792)

##### Notes

Siberic-European. Paludicolous. Macropterous, with summer larvae. Small size. Predator.

Rare in the study area (*n* = 15).

#### Ophonus azureus

(Fabricius, 1775)

##### Notes

Central Asiatic-European-Mediterranean. Open habitats, xerophilous. Pteridimorphic, with winter larvae. Small size. Spermatophagous.

Rare in the study area (*n* = 1); recorded in herbaceous buffer strips only.

#### Ophonus cribricollis

(Dejean, 1829)

##### Notes

Turanic-European. Open habitats, xerophilous. Macropterous, with winter larvae. Small size. Spermatophagous.

Rare in the study area (*n* = 2); recorded in rice field banks only.

#### Ophonus diffinis

(Dejean, 1829)

##### Notes

European. Open habitats. Macropterous, with winter larvae. Medium size. Spermatophagous.

Rare in the study area (*n* = 1); recorded in herbaceous restored habitats only.

#### Ophonus parallelus

(Dejean, 1829)

##### Notes

European. Open habitats. Macropterous, with winter larvae. Small size. Spermatophagous.

Rare in the study area (*n* = 3); recorded in arboreal restored habitats only.

#### Panagaeus cruxmajor

(Linné, 1758)

##### Notes

Siberic-European. Open habitats, hygrophilous. Macropterous, with summer larvae. Small size.

Rare in the study area (*n* = 1); recorded in herbaceous buffer strips only.

#### Paranchus albipes

(Fabricius, 1796)

##### Notes

European-Mediterranean (Holoartic). Ripicolous. Macropterous, with summer larvae. Small size.

Rare in the study area (*n* = 1); recorded in arboreal restored habitats only.

#### Parophonus hirsutulus

(Dejean, 1829)

##### Notes

Turanic-Mediterranean. Open habitats, hygrophilous. Macropterous, with summer larvae. Small size.

Common in the study area (*n* = 190). Recorded in all habitat categories.

#### Parophonus maculicornis

(Duftschmid, 1812)

##### Notes

S-European. Open habitats, thermophilous. Macropterous, with summer larvae. Small size. Zoospermatophagous.

Common in the study area (*n* = 65).

#### Parophonus mendax

(P. Rossi, 1790)

##### Notes

S-European. Open habitats, thermophilous. Macropterous, with summer larvae. Small size.

Uncommon north of the Po river. Rare in the study area (*n* = 18).

#### Parophonus planicollis

(Dejean, 1829)

##### Notes

E-Mediterranean. Open habitats, thermophilous. Macropterous, with summer larvae. Small size. Zoospermatophagous.

Uncommon north of the Po river. Rare in the study area (*n* = 11).

#### Patrobus atrorufus

(Stroem, 1768)

##### Notes

Siberic-European. Silvi-ripicolous. Ptedirimorphic, with winter larvae. Small size. Predator.

Common in the study area (*n* = 314).

#### Philochthus lunulatus

(Geffroy in Fourcroy, 1785)

##### Notes

European-Mediterranean. Open habitats, hygrophilous. Macropterous, with summer larvae. Very small size. Predator.

Rare in the study area (*n* = 28). Recorded in all habitat categories.

#### Poecilus cupreus

(Linné, 1758)

##### Notes

Asiatic-European. Open habitats, eurytopic. Macropterous, with summer larvae. Medium size. Zoospermatophagous.

Dominant in the study area (*n* = 6127). Recorded in all habitat categories.

#### Poecilus versicolor

(Sturm, 1824)

##### Notes

Asiatic-European. Open habitats, hygrophilous. Macropterous, with summer larvae. Medium size. Predator.

Common in the study area (*n* = 1025). Recorded in all habitat categories.

#### Pseudoophonus griseus

(Panzer, 1796)

##### Notes

Paleartic. Open habitats, eurytopic. Macropterous, with winter larvae. Medium size.

Common in the study area (*n* = 286). Recorded in all habitat categories.

#### Pseudoophonus rufipes

(De Geer, 1774)

##### Notes

Paleartic (Holoartic). Open habitats, eurytopic. Macropterous, with winter larvae. Medium size. Zoospermatophagous.

Dominant in the study area (*n* = 12 626). Recorded in all habitat categories.

#### Pterostichus aterrimus

(Herbst, 1784)

##### Notes

W-Paleartic. Paludicolous, silvi-ripicolous. Macropterous, with summer larvae. Medium size.

Rare in the study area (*n* = 25). Recorded in all habitat categories.

#### Pterostichus macer

(Marsham, 1802)

##### Notes

Asiatic-European. Open habitats, xerophilous. Macropterous, with summer larvae. Medium size. Predator.

Uncommon north of the Po river. Rare in the study area (*n* = 1); recorded in rice field banks only.

#### Pterostichus melanarius

(Illiger, 1798)

##### Notes

Holoartic. Eurytopic. Pteridimorphic, with winter larvae. Large size. Predator.

Common in the study area (*n* = 869). Recorded in all habitat categories.

#### Pterostichus niger

(Schaller, 1783)

##### Notes

Asiatic-European. Silvicolous, hygrophilous. Pteridimorphic, with winter larvae. Large size. Predator.

Common in the study area (*n* = 1292). Recorded in all habitat categories.

#### Pterostichus nigrita

(Paykull, 1790)

##### Notes

Paleartic. Eurytopic, hygrophilus. Pteridimorphic, with summer larvae. Medium size. Predator.

Rare in the study area (*n* = 34).

#### Pterostichus strenuus

(Panzer, 1797)

##### Notes

Asiatic-European. Silvi-ripicolous. Pteridimorphic, with summer larvae. Small size. Predator.

Common in the study area (*n* = 263). Recorded in all habitat categories.

#### Pterostichus vernalis

(Panzer, 1796)

##### Notes

Paleartic. Eurytopic, hygrophilous. Macropterous, with summer larvae. Small size. Predator.

Common in the study area (*n* = 160). Recorded in all habitat categories.

#### Sphaerotachys hoemorrhoidalis

(Ponza, 1805)

##### Notes

Afrotropical-Mediterranean. Open habitats, hygrophilous. Macropterous, with summer larvae. Very small size.

Rare in the study area (*n* = 4).

#### Stenolophus mixtus

(Herbst, 1784)

##### Notes

Paleartic. Paludicolous. Macropterous, with summer larvae. Small size. Zoospermatophagous.

Rare in the study area (*n* = 5).

#### Stenolophus teutonus

(Schrank, 1781)

##### Notes

Turanic-European-Mediterranean. Open habitats, hygrophilous. Macropterous, with summer larvae. Small size.

Common in the study area (*n* = 605). Recorded in all habitat categories.

#### Syntomus obscuroguttatus

(Duftschmid, 1812)

##### Notes

European-Mediterranean. Eurytopic. Macropterous, with summer larvae. Very small size. Predator.

Common in the study area (*n* = 190).

#### Syntomus truncatellus

(Linné, 1761)

##### Notes

Siberic-European. Silvicolous. Pteridimorphic, with summer larvae. Very small size. Predator.

Rare in the study area (*n* = 8).

#### Synuchus vivalis

(Illiger, 1798)

##### Notes

Asiatic-European. Silvicolous, hygrophilous. Pteridimorphic, with winter larvae. Small size. Zoospermatophagous.

Rare in the study area (*n* = 1); recorded in arboreal restored habitats only.

#### Trechus quadristriatus

(Schrank, 1781)

##### Notes

Turanic-European-Mediterranean. Eurytopic. Pteridimorphic, with winter larvae. Very small size. Predator.

Rare in the study area (*n* = 7).

## Analysis

Overall, we collected 34,108 individuals belonging to 98 carabid species. We recorded 65 species in rice field banks, 73 species in buffer strips and 78 in restored habitats. Eight species were found only in rice field banks (*Agonum sexpunctatum*, *Amara nitida*, *Cicindela campestris*, *Clivina collaris*, *Harpalus albanicus*, *Harpalus pumilus*, *Ophonus cribricollis*, *Pterostichus macer*), 6 species only in herbaceous buffer strips (*Amara littorea*, *Bembidion quadripustulatum*, *Bradycellus verbasci*, *Dolichus halensis*, *Ophonus azureus*, *Panagaeus cruxmajor*), 2 species only in arboreal buffer strips (*Badister sodalis*, *Drypta dentata*), 2 species only in herbaceous restored habitats (*Acupalpus elegans*, *Ophonus diffinis*) and 11 species only in arboreal restored habitats (*Acinopus picipes*, *Acupalpus flavicollis*, *Acupalpus notatus*, *Agonum versutum*, *Agonum viduum*, *Limodromus assimilis*, *Limodromus krynickii*, *Nebria brevicollis*, *Ophonus parallelus*, *Paranchus albipes*, *Synuchus vivalis*). *Poecilus cupreus* and *Pseudoophonus rufipes* consituted about 55% of the capture with 18 753 individuals.

**Figure 5.**
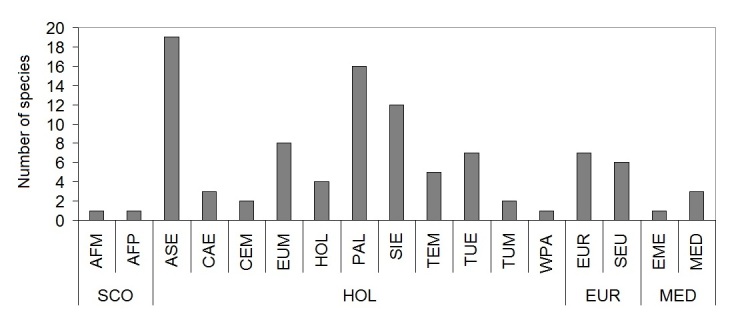
Chorotypes of ground beetles collected in the study area during 2009 and 2010 (AFM = Afrotropical-Mediterranean, AFP = Afrotropical and Paleartic, ASE = Asiatic-European, CAE = Central Asiatic-European, CEM = Central Asiatic-European-Mediterranean, EME = E-Mediterranean, EUM = European-Mediterranean, EUR = European, MED = Mediterranean, HOL = Holoartic, PAL = Paleartic, SCO = Subcosmopolitan, SEU = S-European, SIE = Siberic-European, TEM = Turanic-European-Mediterranean, TUE = Turanic-European, TUM = Turanic-Mediterranean, WPA = W-Paleartic) (plotted after data in Table 1).

The collected species belonged to 17 chorotypes (Fig. 5), grouped into 4 complexes (Subcosmopolitan, Holoartic, European and Mediterranean). About 80% of the species captured in the area were Holoartic, 13.3% European, 4.1% Mediterranean and 2% Subcosmopolitan (Table 1). Most of the species were small (very small species: 18.4%, small species: 46.9%) and medium (28.6%); only 6.1% of the captured carabids had size larger than 15 mm (*Calosoma auropunctatum*, *Carabus granulatus*, *Chlaenius spoliatus*, *Dolichus halensis*, *Pterostichus melanarius* and *Pterostichus niger*). About 80% of the collected species had larvae that develop during summer, without dormancy (i.e. were spring breeders) and 18.4% were species with winter larvae, that grow slowly with compulsory dormancy (i.e. were autumn breeders). *Cicindela campestris* (one individual recorded along rice field banks) was the only species with poliennal larvae. Macropterous and pteridimorphic species were 82.7% and 17.3% respectively; we didn’t find any strictly brachypterous species.

**Figure 6.**
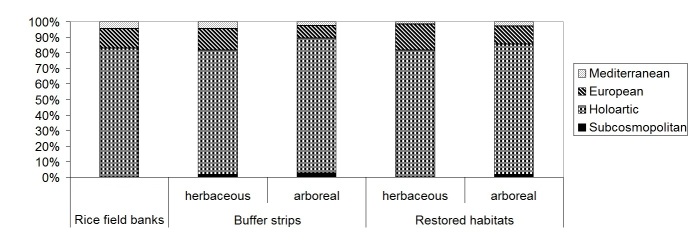
Percentage of carabid species for each chorological complexes (Subcosmopolitan, Holoartic, European, Mediterranean) in rice field banks, buffer strips and restored habitats (plotted after data in Table 1).

**Figure 7.**
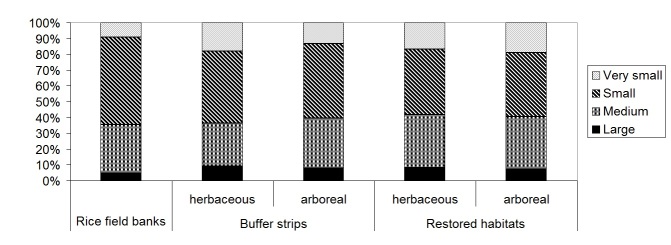
Percentage of carabid species for each body size (very small: < 5 mm, small: 5 – 9 mm, medium: 9 – 15 mm, large: > 15 mm) in rice field banks, buffer strips and restored habitats (plotted after data in Table 1).

**Figure 8.**
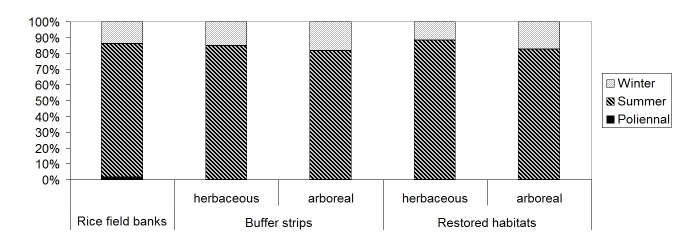
Percentage of carabid species for each larval development (summer, winter, poliennal) in rice field banks, buffer strips and restored habitats (plotted after data in Table 1).

**Figure 9.**
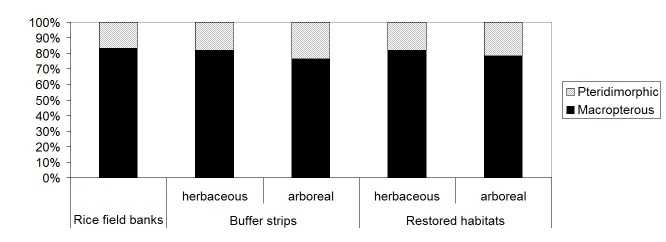
Percentage of carabid species for each wing development (macropterous and pteridimorphic) in rice field banks, buffer strips and restored habitats (plotted after data in Table 1).

Also rice field banks, buffer strips and restored habitats, analyzed separately, were dominated by Holoartic, medium-small, winged species, with summer larvae (Table 1); species number and percentages for chorotype, body size, larval and wing development were similar in the different habitat categories (Figs 6, 7, 8, 9).

## Discussion

On the whole, 98 carabid species were collected in rice field banks, buffer strips adjacent to paddy fields, and restored habitats (herbaceous and arboreal). Species number could be slightly underestimated because of the sampling method which is not very well suited for some taxa as Lebiinae and Bembidinae. Nevertheless, the area resulted species-rich, especially when you consider that it is not placed inside a riverine corridor and when you compare the species number with that recorded in other anthropogenic habitats of the Po plain: 60-70 species in rye, oat and fallow fields (Pescarolo 1990, Pescarolo 1993); 48 species in a complex of habitats composed by one poplar grove, one artificial wetland, banks of irrigation canals and cropped areas (Casale et al. 1993); 55 species in poplar groves of different ages (Casale et al. 1993); 60 species in meadows of different ages (Gobbi et al. 2005); 60 species in meadows, crops and reforested areas of two urban parks in Milan (Pilon et al. 2010).

Most of the collected carabids, both in the whole area and in each habitat categories, were species with a wide distribution in the Paleartic region, eurytopic and common in European agroecosystems. The assemblages were dominated by small-medium, macropterous species, with summer larvae; we didn’t find any endemism.

No brachypterous and strictly forest-dwelling species were sampled, despite the presence of some recent woodlots (i.e about 10 years old). In fact, species unable to disperse by flight were prevented to colonize these stands (including *Abax continuus* Ganglbauer 1891, very common in woods of the Lombardy plain), because of the absence of ecological corridors connecting woodlots with forest remnants (Macarthur and Wilson 1967). As a consequence, the Carabid fauna was mainly composed by species of open habitats. Most of the species were also hygrophilous, due to a dense network of artificial irrigation canals and a superficial water-table.

The most interesting aspect of this Carabid coenosis is the presence of several species with southern distribution, quite common in clay soil on the right bank of the Po river, and known only in few stations north of the Po river. Among these species, we list *Acinopus picipes*, *Amblystomus niger*, *Dinodes decipiens*, *Harpalus cupreus*, *Harpalus oblitus*, *Parophonus mendax*, *Parophonus planicollis*, *Pterostichus macer*. Although a comparison with the past coenosis is not possible for the lack of similar surveys in the area, it could be hypothesized that these species are recent colonizers (7-10 years). They are not reported in the historical catalogue of Magistretti (1965), and are also not listed in several recent faunistic investigations carried out in the Lombardy lowland, particularly along the Ticino river (Pasquetto 1992, Bogliani et al. 2003), Adda river (Conti 1991), Po river (Pilon et al. 1991, Rancati and Sciaky 1994) and in Milan (Pilon et al. 2010), where potentially suitable habitats were sampled. Even in an intensive survey along the Po river included in Piedmont region, only some of these species have been collected (Allegro and Sciaky 2001). If so, we could assume a tendency to a northward shift in the distribution of these species, according to what has been observed for other zoological groups well studied and that have great mobility, such as birds (Chen et al. 2011) and dragonflies (Ott 2010).

We underline also the presence of *Brachinus plagiatus*, an uncommon halophilous species. Moreover *Amara littorea*, an Asiatic-European distribution species, has been recorded with certainty for the first time in Italy (Cardarelli and Pilon 2012).

## Supplementary Material

XML Treatment for Acinopus picipes

XML Treatment for Acupalpus elegans

XML Treatment for Acupalpus flavicollis

XML Treatment for Acupalpus maculatus

XML Treatment for Acupalpus notatus

XML Treatment for Agonum emarginatum

XML Treatment for Agonum muelleri

XML Treatment for Agonum sexpunctatum

XML Treatment for Agonum versutum

XML Treatment for Agonum viduum

XML Treatment for Amara aenea

XML Treatment for Amara bifrons

XML Treatment for Amara communis

XML Treatment for Amara familiaris

XML Treatment for Amara fulvipes

XML Treatment for Amara littorea

XML Treatment for Amara lucida

XML Treatment for Amara nitida

XML Treatment for Amara similata

XML Treatment for Amblystomus niger

XML Treatment for Anchomenus dorsalis

XML Treatment for Anisodactylus binotatus

XML Treatment for Anisodactylus signatus

XML Treatment for Badister bullatus

XML Treatment for Badister sodalis

XML Treatment for Bembidion quadrimaculatum

XML Treatment for Bembidion quadripustulatum

XML Treatment for Brachinus elegans

XML Treatment for Brachinus explodens

XML Treatment for Brachinus glabratus

XML Treatment for Brachinus plagiatus

XML Treatment for Brachinus sclopeta

XML Treatment for Bradycellus verbasci

XML Treatment for Calathus fuscipes

XML Treatment for Calathus melanocephalus

XML Treatment for Calosoma auropunctatum

XML Treatment for Carabus granulatus

XML Treatment for Chlaeniellus nitidulus

XML Treatment for Chlaeniellus tristis

XML Treatment for Chlaenius spoliatus

XML Treatment for Cicindela campestris

XML Treatment for Clivina collaris

XML Treatment for Clivina fossor

XML Treatment for Diachromus germanus

XML Treatment for Dinodes decipiens

XML Treatment for Dolichus halensis

XML Treatment for Drypta dentata

XML Treatment for Harpalus affinis

XML Treatment for Harpalus albanicus

XML Treatment for Harpalus anxius

XML Treatment for Harpalus cupreus

XML Treatment for Harpalus dimidiatus

XML Treatment for Harpalus distinguendus

XML Treatment for Harpalus luteicornis

XML Treatment for Harpalus oblitus

XML Treatment for Harpalus pumilus

XML Treatment for Harpalus pygmaeus

XML Treatment for Harpalus rubripes

XML Treatment for Harpalus serripes

XML Treatment for Harpalus tardus

XML Treatment for Limodromus assimilis

XML Treatment for Limodromus krynickii

XML Treatment for Metallina lampros

XML Treatment for Metallina properans

XML Treatment for Microlestes corticalis

XML Treatment for Microlestes minutulus

XML Treatment for Nebria brevicollis

XML Treatment for Oodes helopioides

XML Treatment for Ophonus azureus

XML Treatment for Ophonus cribricollis

XML Treatment for Ophonus diffinis

XML Treatment for Ophonus parallelus

XML Treatment for Panagaeus cruxmajor

XML Treatment for Paranchus albipes

XML Treatment for Parophonus hirsutulus

XML Treatment for Parophonus maculicornis

XML Treatment for Parophonus mendax

XML Treatment for Parophonus planicollis

XML Treatment for Patrobus atrorufus

XML Treatment for Philochthus lunulatus

XML Treatment for Poecilus cupreus

XML Treatment for Poecilus versicolor

XML Treatment for Pseudoophonus griseus

XML Treatment for Pseudoophonus rufipes

XML Treatment for Pterostichus aterrimus

XML Treatment for Pterostichus macer

XML Treatment for Pterostichus melanarius

XML Treatment for Pterostichus niger

XML Treatment for Pterostichus nigrita

XML Treatment for Pterostichus strenuus

XML Treatment for Pterostichus vernalis

XML Treatment for Sphaerotachys hoemorrhoidalis

XML Treatment for Stenolophus mixtus

XML Treatment for Stenolophus teutonus

XML Treatment for Syntomus obscuroguttatus

XML Treatment for Syntomus truncatellus

XML Treatment for Synuchus vivalis

XML Treatment for Trechus quadristriatus

## Figures and Tables

**Table 1.** Number and percentage of carabid species for each ecological categories (chorological complexes, body size, larval and wing development) in rice field banks, buffer strips and restored habitats.

	Rice field banks		Buffer strips				Restored habitats				Total	
			herbaceous		arboreal		herbaceous		arboreal			
	N	%	N	%	N	%	N	%	N	%	N	%
**Chorotype**												
Subcosmopolitan	0	0	1	1.5	1	2.6	0	0	1	1.4	2	2
Holoartic	54	83.1	53	80.3	33	86.8	49	81.7	58	84.1	79	80.6
European	8	12.3	9	13.7	3	7.9	10	16.7	8	11.6	13	13.3
Mediterranean	3	4.6	3	4.5	1	2.7	1	1.6	2	2.9	4	4.1
**Size**												
Very small	6	9.2	12	18.2	5	13.2	10	16.7	13	18.8	18	18.4
Small	36	55.4	30	45.5	18	47.4	25	41.7	28	40.6	46	46.9
Medium	20	30.8	18	27.2	12	31.5	20	33.3	23	33.3	28	46.9
Large	3	4.6	6	9.1	3	7.9	5	8.3	5	7.3	6	6.1
**Larvae**												
summer	55	84.6	56	84.8	31	81.6	53	88.3	57	82.6	79	80.6
winter	9	13.8	10	15.2	7	18.4	7	11.7	12	17.4	18	18.4
poliennal	1	1.5	0	0.0	0	0.0	0	0.0	0	0.0	1	1.0
**Wing**												
macropterous	54	83.1	54	81.8	29	76.3	49	81.7	54	78.3	81	82.7
pteridimorphic	11	16.9	12	18.2	9	23.7	11	18.3	15	21.7	17	17.3

## Supplementary files

### Frequency (N) and annual Activity Density (AD) of collected species in rice field banks, buffer strips and restored habitats during 2009 and 2010

**Authors:** Nicola Pilon, Elisa Cardarelli, Giuseppe Bogliani **Data type:** occurrences In 2009 and 2010, ground beetles (ColeopteraCarabidae) were sampled in an agricultural area of the Po plain (Lombardy, Italy), by means of pitfall traps. The dataset reported frequency (N) and annual Activity Density (AD) of collected species in: (a) rice field banks; (b) herbaceous buffer strips; (c) arboreal buffer strips; (d) herbaceous restored habitats, i.e. wet and dry meadows; (e) arboreal restored habitats, i.e. forested areas and hedges. **File:** Pilon_Carabidae_SupplementaryFile.xls

## References

[B184478] Allegro Gianni, Sciaky Riccardo (2001). I Coleotteri Carabidi del Po piemontese (tratto orientale). Bollettino del Museo Regionale di Scienze Naturali, Torino,.

[B378762] Bettacchioli Giovanni, Taormina Mauro, Bernini Fabio, Migliorini Massimo (2012). Disturbance regimes in a wetland remnant: implications for trait-displacements and shifts in the assemblage structure of carabid beetles (Coleoptera: Carabidae). Journal of Insect Conservation.

[B184540] Bogliani Giuseppe, Bontardelli Laura, Giordano Valentina, Lazzarini Monica, Rubolini Diego (2003). Biodiversità animale degli ambienti terrestri nei parchi del Ticino.

[B184390] Brandmayr Pietro, Zetto Tullia, Pizzolotto Roberto (2005). I Coleotteri Carabidi per la valutazione ambientale e la conservazione della biodiversità.

[B184391] Cardarelli Elisa, Pilon Nicola (2012). Segnalazioni faunistiche italiane. Amara littorea. Bollettino della Società Entomologica Italiana.

[B378641] Casale A., Sturani M., Vigna-Taglianti A. (1982). Fauna d'Italia 18. ColeopteraCarabidae 1. Introduzione, Paussinae, Carabinae.

[B378717] Casale A., Giachino P. M., Allegro G., Beffa G. Della, Picco F. (1993). Comunita` di Coleotteri Carabidi (Coleoptera) in pioppeti del Piemonte meridionale. Rivista Piemontese di Storia Naturale.

[B184551] Chen I. -C., Hill J. K., Ohlemuller R., Roy D. B., Thomas C. D. (2011). Rapid Range Shifts of Species Associated with High Levels of Climate Warming. Science.

[B378589] Cole L. J., McCracken D. I., Dennis P., Downie I. S., Griffin A. L., Foster G. N., Murphy K. J., Waterhouse T. (2002). Relationships between agricultural management and ecological groups of ground beetles (Coleoptera: Carabidae) on Scottish farmland. Agriculture, Ecosystems and Environment.

[B184617] Conti Elisabetta (1991). Cenosi carabidologiche del Parco Adda Sud, Zelo Buon Persico, Milano.

[B378571] Drioli G. (1987). Tipi e tempi di sviluppo dei coleotteri geoadefagi presenti sul basso carso triestino.

[B184506] Fournier Elisabeth, Loreau Michel (1999). Effects of newly planted hedges on ground-beetle diversity (Coleoptera, Carabidae) in an agricultural landscape. Ecography.

[B378728] Gobbi M., Fontaneto D., Guidali F. (2005). Carabid beetles (InsectaColeoptera) in meadows in Lombardia (Italy) lowland. Annali del Museo Civico di Storia Naturale di Ferrara.

[B184479] Hůrka Karel (1996). Carabidae of the Czech and Slovak Republics.

[B378530] Jeannel R. (1941). Faune de France 39. Coleoptere Carabiques 1.

[B378562] Jeannel R. (1942). Faune de France 40. Coleoptere Carabiques 2.

[B184534] Kleijn David, Sutherland William J. (2003). How effective are European agri-environment schemes in conserving and promoting biodiversity?. Journal of Applied Ecology.

[B184482] Kleijn David, Berendse Frank, Smit Ruben, Gilissen Niels (2001). Agri-environment schemes do not effectively protect biodiversity in Dutch agricultural landscapes. Nature.

[B309003] Region Lombardy (2012). Piano di Sviluppo Rurale (Rural Development Plan) 2007-2013. Asse 2. Miglioramento dell'ambiente e dello spazio rurale. http://www.agricoltura.regione.lombardia.it.

[B184485] Macarthur Robert H., Wilson Edward O. (1967). The Theory of Island Biogeography.

[B184567] Magistretti Mario (1965). Cicindelidae, Carabidae. Catalogo topografico. Fauna d'Italia, Volume 8.

[B378614] Melis Claudia, Olsen Camilla Bjerk, Hyllvang Maria, Gobbi Mauro, Stokke Bård G., Røskaft Eivin (2010). The effect of traffic intensity on ground beetle (Coleoptera: Carabidae) assemblages in central Sweden. Journal of Insect Conservation.

[B184573] Ott Jürgen (2010). Monitoring Climatic Change With Dragonflies.

[B184608] Pasquetto Renata (1992). Indagine eco-faunistica su popolazioni di Coleotteri Carabidi in alcuni biotopi del medio corso del Ticino.

[B184493] Peach Will J, Lovett Lucy J, Wotton Simon R, Jeffs Cath (2001). Countryside stewardship delivers cirl buntings (Emberiza cirlus) in Devon, UK. Biological Conservation.

[B378697] Pescarolo R. (1990). Ricerche sui coleotteri della valle del Ticino. Rivista Piemontese di Storia Naturale.

[B378707] Pescarolo R. (1993). I coleotteri carabidi della baraggia di Piano Rosa (Piemonte, Novara). Rivista Piemontese di Storia Naturale.

[B184406] Pilon Nicola, Sciaky Riccardo, Violani Carlo (1991). La carabidofauna di un biotopo ripario del corso lombardo del Po (ColeopteraCarabidae). Memorie della Società entomologica italiana, Genova,.

[B184433] Pilon Nicola, Zoia Stefano, Trotta Alessio (2010). Artropodofauna dei parchi milanesi Boscoincittà e Parco delle Cave (Araneae; ColeopteraCarabidae, Staphylinidae, Leiodidae). Atti della Società italiana di Scienze naturali e del Museo civico di Storia naturale di Milano,.

[B378629] Purtauf Tobias, Dauber Jens, Wolters Volkmar (2005). The response of carabids to landscape simplification differs between trophic groups. Oecologia.

[B184459] Rancati Stefano, Sciaky Riccardo (1994). Analisi delle carabidocenosi presenti in alcuni biotopi golenali del Po (Cremona). Pianura, Supplemento di “Provincia Nuova”, Cremona,.

[B184579] Reid Neil, McDonald Robbie A., Montgomery W. Ian (2007). Mammals and agri-environment schemes: hare haven or pest paradise?. Journal of Applied Ecology.

[B184517] Stoate C, Boatman N. D, Borralho R. J, Carvalho C. Rio, Snoo G. R.de, Eden P (2001). Ecological impacts of arable intensification in Europe. Journal of Environmental Management.

[B184581] Stoate C., Báldi A., Beja P., Boatman N. D., Herzon I., Doorn A. van, Snoo G. R. de, Rakosy L., Ramwell C. (2009). Ecological impacts of early 21st century agricultural change in Europe – A review.. Journal of Environmental Management.

[B184450] Vickery Juliet A, Bradbury Richard B, Henderson Ian G, Eaton Mark A, Grice Philip V (2004). The role of agri-environment schemes and farm management practices in reversing the decline of farmland birds in England. Biological Conservation.

[B184386] Vigna-Taglianti Augusto, Brandmayr P, Zetto T, Pizzolotto R (2005). I Coleotteri Carabidi per la valutazione ambientale e la conservazione della biodiversità.

[B309044] Vigna-Taglianti A (2010). Fauna Europea: Carabidae. http://www.faunaeur.org.

